# Periodic and transient motions of large woodpeckers

**DOI:** 10.1038/s41598-017-13035-6

**Published:** 2017-10-02

**Authors:** Michael D. Collins

**Affiliations:** Naval Research Laboratory, Washington, D.C, 20375 USA

## Abstract

Two types of periodic and transient motions of large woodpeckers are considered. A drumming woodpecker may be modeled as a harmonic oscillator with a periodic forcing function. The transient behavior that occurs after the forcing is turned off suggests that the double knocks of *Campephilus* woodpeckers may be modeled in terms of a harmonic oscillator with an impulsive forcing, and this hypothesis is consistent with audio and video recordings. Wingbeats are another type of periodic and transient motion of large woodpeckers. A model for the flap rate in cruising flight is applied to the Pileated Woodpecker (*Dryocopus pileatus*) and the Ivory-billed Woodpecker (*Campephilus principalis*). During a brief transient just after taking off, the wing motion and flap rate of a large woodpecker may not be the same as in cruising flight. These concepts are relevant to videos that contain evidence for the persistence of the Ivory-billed Woodpecker.

## Introduction

Drumming and wingbeats are two types of periodic motion of large woodpeckers. This paper discusses these behaviors and related transient motions that occur in evidence for the persistence of the Ivory-billed Woodpecker (*Campephilus principalis*)^1–3^. Most woodpeckers signal by drumming, which consists of a rapid series of blows with the bill. Audio S1 contains four drumming events by nearby and distant Pileated Woodpeckers (*Dryocopus pileatus*). Some members of the *Campephilus* genus do not engage in this type of drumming but instead signal with double knocks. Audio S2 contains double knocks by five *Campephilus* woodpecker species. It is proposed that both of these behaviors may be modeled in terms of a harmonic oscillator^4^, and evidence is presented to support the hypothesis that double knocks correspond to a special case of drumming in which the driving force is impulsive rather than periodic, as it is in the case of most woodpeckers. The flap rate of a bird in cruising flight is amenable to statistical analysis^5–7^. In putative video footage of an Ivory-billed Woodpecker in cruising flight (Movie S5 of ref.^3^), the flap rate is about ten standard deviations greater than the mean flap rate of the Pileated Woodpecker. In a test of the consistency of this evidence, a flap rate model based on vortex shedding^8,9^ is applied to the Pileated and Ivory-billed Woodpeckers. Large woodpeckers fold their wings closed during the middle of the upstroke during cruising flight;^7^ an example of this behavior is shown in Movie S7 of ref.^3^. During the first several flaps after taking off, there may be a transient in which the wing motion and flap rate are not the same as in cruising flight.

## Methods

### Model for a Drumming Woodpecker

A drumming woodpecker may be modeled as a harmonic oscillator, which is a mechanical system in which an object experiences a restoring force when it is displaced from an equilibrium point. The object may have periodic or transient motions, depending on how it is forced. In the following example of a harmonic oscillator^4^,

1 $$\frac{{d}^{2}x}{d{t}^{2}}+p\frac{dx}{dt}+qx=R(t),$$

**Figure 1:**
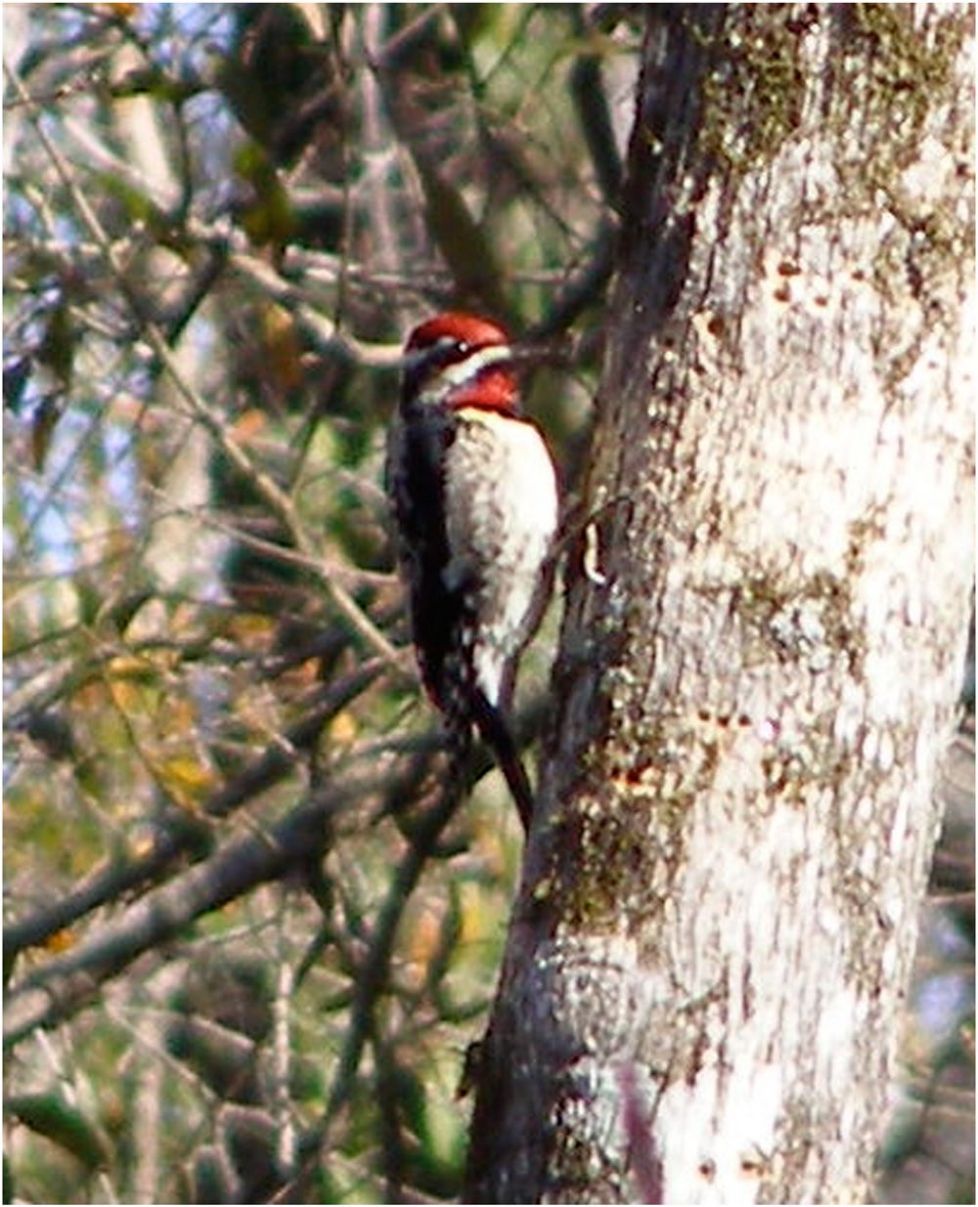
A perched Yellow-bellied Sapsucker (*Sphyrapicus varius*) using its feet and tail as anchor points. This image was obtained in the Pearl River swamp in Louisiana by the author.

*x* is the displacement, *t* is time, the coefficient *p* is related to damping (energy loss), the coefficient *q* is related to the natural frequency of the system, and *R*(*t*) is the forcing function. As shown in Fig. 1, a woodpecker uses its feet and tail as anchor points when perched upright. Tension through the neck, body, and tail is a restoring force that accelerates the head and bill toward the wood, where the bill rebounds with a loss of energy after each impact. Drumming is driven by periodic oscillations of the body that are tuned to the natural frequency range of the system. When the bill impacts the wood at *x* = *x*_0_, it rebounds with a loss of energy that can be modeled with the change in velocity,

2 $$\frac{dx}{dt}\to -\alpha \frac{dx}{dt},$$

where *α* < 1. In order to illustrate some of the basic behaviors of harmonic oscillators, we consider the case *p* = 0.2, *q* = 0.3, *α*** = **0.8, and *x*_0_ = 0.2, with the periodic forcing function,

3 $$R(t)=\{\begin{array}{cc}\sin (t) & 2\pi  < t < 20\pi \\ 0 & {\rm{o}}{\rm{t}}{\rm{h}}{\rm{e}}{\rm{r}}{\rm{w}}{\rm{i}}{\rm{s}}{\rm{e}}\end{array}$$

and the impulsive forcing function,

4 $$R(t)=\exp [-{(t-20\pi )}^{2}].$$

**Figure 2:**
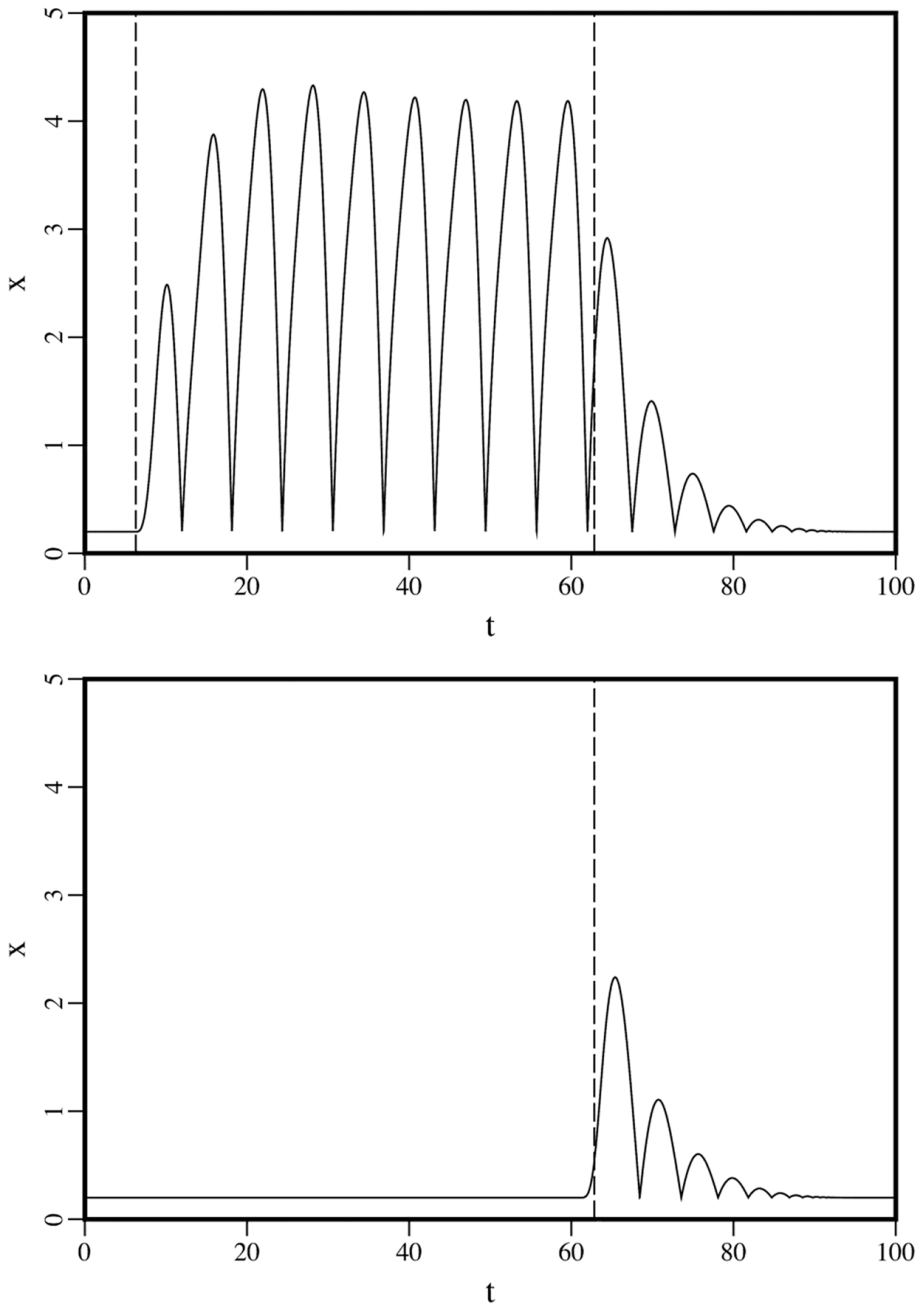
Solutions of Eq. (1) corresponding to periodic (top) and impulsive (bottom) forcing functions. The vertical dashed lines indicate the points when the periodic driving force is turned on and off and when the impulsive driving force reaches its maximum amplitude.

Solutions for these cases appear in Fig. 2. For the periodic case, there are transients after the forcing function is turned on at *t* = 2*π* and after the forcing function is turned off at *t* = 20*π*, with a periodic response between the transients. For the impulsive case, the response is a transient that is similar to the transient that follows the periodic forcing.

### Model for Flap Rate in Cruising Flight

The flap rate *f* of a bird in cruising flight is amenable to statistical analysis and limited to a narrow range of frequencies for each species. For most species that have been studied, the standard deviation of the flap rate has been found to be less than ten percent of the mean flap rate^5–7^. The flap rate model^8,9^,

5 $$f=\frac{St\cdot U}{b\,\sin (33.5{b}^{-0.24})},$$

is based on vortex shedding, where *St* is the Strouhal number, *U* is the flight speed, and *b* is the wingspan. Large woodpeckers have a flap style in which the wings are folded closed and briefly paused during the middle of each upstroke^7^. For such intermittent flight, an appropriate value for the Strouhal number is 0.25^9^, and the period of a flap corresponds to the time between when the wings begin to open after a pause and when they close at the beginning of the next pause.

## Results

### Periodic and Transient Drumming

As discussed in Movie S1, oscillations of the body of a drumming woodpecker are visible along the back and tail. As would be expected for a damped harmonic oscillator with a periodic driving force, there is a transient consisting of a few additional impacts after the driving force is turned off; this is analogous to the transient that follows the periodic response in the upper part of Fig. 2. There are no additional impacts after the tension in the body (the restoring force) is released and the bird pulls its head back from the tree. The behavior of this system suggests the hypothesis that double knocks by *Campephilus* woodpeckers correspond to a special case in which the driving force of the harmonic oscillator is impulsive rather than periodic, with the impacts analogous to the transient that occurs after the periodic driving force of a drumming woodpecker is turned off and the transient in the lower part of Fig. 2.

According to this hypothesis, the time interval between impacts and the number of impacts that would be detected at a distance should depend on the amount of tension in the body, the intensity of the impulsive driving force, the properties of the wood, and the amount of time that elapses before the bird releases the tension in its body. These considerations are consistent with Tanner’s account of the Ivory-billed Woodpecker giving both single and double knocks^10^ (p. 62) and with the recordings of the transient drumming of three *Campephilus* woodpecker species in Audio S3 that consist of more than two impacts of decreasing amplitude. During a double knock by a Pale-billed Woodpecker (*Campephilus guatemalensis*) that appears in Movie S2, there is only one thrust of the body, and the second impact occurs after the bill rebounds from the wood. Several double knocks of the Magellanic Woodpecker (*Campephilus magellanicus*) appear in a documentary by David Attenborough^11,12^, and each of them results from a single thrust of the body. In putative video footage of an Ivory-billed Woodpecker giving a double knock (Movie S8 of ref.^3^), there is only one thrust of the body. These examples suggest that a double knock is the result of a single thrust rather than a pair of deliberate blows that are given in rapid succession.

### Periodic and Transient Flaps

For birds that have intermittent flaps, the raw flap rate $\mathop{f}\limits^{ \sim }$ is defined to be the number of flaps divided by the elapsed time. The intrinsic flap rate *f*, which appears in Eq. (5), is determined by factoring out intervals in which the wings are held fixed. The ratio $\mathop{f}\limits^{ \sim }/f$ corresponds to the fraction of cycle time spent flapping, which is 0.714 for the Pileated Woodpecker^7^. It is possible to obtain an accurate estimate of $\mathop{f}\limits^{ \sim }$ by averaging over several flaps (the only potential sources of error are the estimates of when the first flap begins and the final flap ends), but this approach cannot be used to directly estimate *f* for a species with intermittent flaps (errors may be introduced in the estimates of when each flap begins and ends). When the bird was flying toward the camera in the cruising flight in Movie S5 of ref.^3^, there were 6 to 7 frames per flap, which corresponds to *f* = 8.5 to 10 Hz. In that part of the video, there were 15 flaps during an interval of 2.27 s^13^, which corresponds to $\mathop{f}\limits^{ \sim }=6.61$ Hz. If the two large woodpeckers have similar values of $\mathop{f}\limits^{ \sim }/f$, we would obtain the estimate *f* = 9.25 Hz, which is consistent with the range of values obtained by counting frames during single flaps.

**Figure 3:**
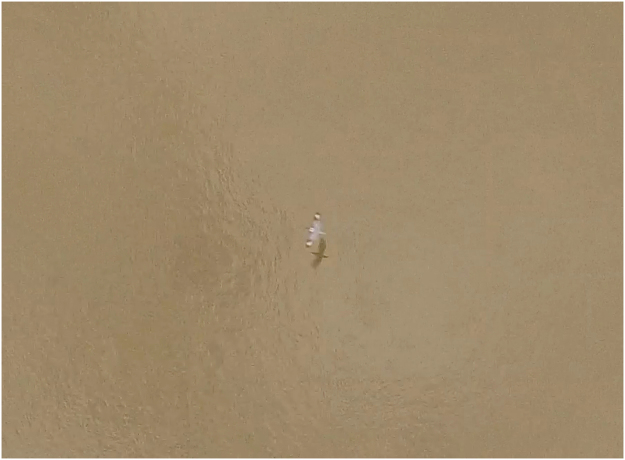
An image of a Belted Kingfisher that was obtained using a drone that was nearly directly above. This image was obtained in the Pearl River swamp in Louisiana by the author.

The bird in the video has a flight speed of 15.2 m/s^13^. In Fig. S2 of ref.^13^, a 0.61 m reference object is compared with the wings of the bird in the video. The wingspan appears to be consistent with the 0.76 to 0.80 m wingspan of the Ivory-billed Woodpecker^14^ but greater than the 0.48 to 0.58 m wingspan of the Belted Kingfisher (*Megaceryle alcyon*)^15^, which is the third largest species of the region (behind the two large woodpeckers) that has a wing motion in which the wings are folded closed (or nearly folded closed) during the flap cycle in cruising flight. As shown in Fig. 3, a Belted Kingfisher that is viewed from above has a white patch on the neck and a distinct pattern of black and white on the outer half of each wing, but there is no trace of these field marks in Movie S5 of ref.^3^. Using the mean value of 0.78 m for the wingspan of the Ivory-billed Woodpecker and 15.2 m/s for the flight speed in Eq. (5), we obtain a predicted flap rate of 8.38 Hz, which differs by 9.4% from the 9.25 Hz flap rate of the bird in the video. Using mean values of 9.55 m/s for the flight speed^7^ and 0.705 m for the wingspan^16^ of the Pileated Woodpecker in Eq. (5), we obtain a predicted flap rate of 5.70 Hz, which differs by 9.6% from the observed value of 5.20 Hz^7^.

During a level takeoff that transitions into cruising flight, there may be a brief transient in which the wing motion and flap rate are not the same as in cruising flight. During the takeoffs by Pileated Woodpeckers that appear in Movie S3, the wings are not folded closed until several flaps after takeoff, and the flap rate decreases from about 7.5 Hz (eight frames per flap) just after takeoff to about 5 Hz (twelve frames per flap) in cruising flight (the latter quantity is consistent with the value given in ref.^7^). In putative video footage of an Ivory-billed Woodpecker taking off (Movie S11 of ref.^3^), the wings are not folded closed and the flap rate is about 10 Hz (six frames per flap) just after takeoff, and this is consistent with an account by Christy of “deep and rapid strokes” at takeoff^17^. Historical accounts and video evidence suggest that the Ivory-billed Woodpecker has higher flap rates than the Pileated Woodpecker during takeoffs and cruising flights. In terms of flap rate, the relationship between these superficially similar species is evidently similar to the relationship between another pair of superficially similar species. Movies S4 and S5 show flights of the Northern Mockingbird (*Mimus polyglottos*) and the Loggerhead Shrike (*Lanius ludovicianus*). Those species are sometimes mistaken for one another when perched, but the difference in flap rate makes it easy to distinguish them immediately after takeoff.

A downward swooping takeoff on fixed wings is an alternative to a level takeoff with deep and rapid flaps. It is known from historical accounts that Ivory-billed Woodpeckers frequently take swooping flights. Putative video footage of swooping takeoffs and landings by Ivory-billed Woodpeckers is presented in Movies S8, S13, S15, S16, S17 and S18 of ref.^3^. During upward swooping landings, woodpeckers typically ascend a short distance and land on a surface that faces the direction of approach. Some of the upward swooping landings in ref.^3^ involve long vertical ascents that provide time for maneuvering and landing on surfaces that do not face the direction of approach. Just after the 2:00 mark in a film of a pair of Magellanic Woodpeckers^11,12^, a close relative of the Ivory-billed Woodpecker, the female flies over to the male and ends the flight with a long vertical ascent that allows time for maneuvering. Just before landing, the bird is ascending and at the same time maneuvering to its left.

## Discussion

Drumming may be modeled as a harmonic oscillator with a periodic forcing. The transient behavior that occurs after the forcing is turned off suggests that double knocks are a transient corresponding to an impulsive forcing that results from a single thrust of the body, and the audio and video examples presented here support this hypothesis. A better understanding of the factors that may cause variations in the number of knocks and the time interval between knocks could be useful in interpreting evidence for the persistence of the Ivory-billed Woodpecker. Flap rate during cruising flight is amenable to statistical analysis, and the flap rate in putative footage of an Ivory-billed Woodpecker in cruising flight is consistent with that species in terms of the prediction of a flap rate model. In cruising flight, the Pileated Woodpecker closes its wings during the middle of the upstroke but uses a different wing motion and a higher flap rate during a transient just after taking off. Video evidence suggests that the Ivory-billed Woodpecker has the same types of wing motion during these types of flight but at higher flap rates.

## Electronic supplementary material

Movie S1

Movie S2

Movie S3

Movie S4

Movie S5

Audio S1

Audio S2

Audio S3

Supplementary information
