# Publisher Correction: Periodic and transient motions of large woodpeckers

**DOI:** 10.1038/s41598-018-25180-7

**Published:** 2018-05-01

**Authors:** Michael D. Collins

**Affiliations:** Naval Research Laboratory, Washington, D.C 20375 USA

Correction to: *Scientific Reports* 10.1038/s41598-017-13035-6, published online 02 October 2017

A supplementary file containing captions for the electronic supplementary material, the movie and audio files, was omitted from the original version of this Article. This has been corrected in the HTML version of the Article; the PDF version was correct at time of publication.

