# Supplementary material for: Periodic and transient motions of large woodpeckers
Source: Sci Rep. 2017 Oct 2;7:12551. doi: 10.1038/s41598-017-13035-6 (PMC5624965; doi:10.1038/s41598-017-13035-6)
Supplement: Supplementary file 9 — Supplementary information [file 41598_2017_13035_MOESM9_ESM.docx]

Audio S1. Audio recording of drumming Pileated Woodpeckers that was obtained by the author in the Pearl River swamp in Louisiana.

Audio S2. Audio recordings from the Macaulay Library (<https://www/macaulaylibrary.org>) at the Cornell Laboratory of Ornithology of double knocks by Pale-billed Woodpecker (*Campephilus guatemalensis*) (ML 215375), Magellanic Woodpecker (*Campephilus magellanicus*) (ML 146641, 164284), Red-necked Woodpecker (*Campephilus rubricollis*) (ML 144593), Cream-backed Woodpecker (*Campephilus leucopogon*) (ML 217073, 213806), and Robust Woodpecker (*Campephilus robustus*) (ML 164265). Narrated by the author. Used with an Open Access license from the Macaulay Library.

Audio S3. Audio recordings from the Macaulay Library (<https://www/macaulaylibrary.org>) at the Cornell Laboratory of Ornithology of transient drumming that consists of more than two knocks by Powerful Woodpecker (*Campephilus pollens*) (ML 58032, 58051), Crimson-crested Woodpecker (*Campephilus melanoleucos*) (ML 77969, 110532), and Crimson-bellied Woodpecker (*Campephilus rubricollis*) (ML 35865). Narrated by the author. Used with an Open Access license from the Macaulay Library.

Movie S1. Discussion by the author of a drumming Pileated Woodpecker that was filmed in the Pearl River swamp in Louisiana by the author.

Movie S2. A Pale-billed Woodpecker giving a double knock that was filmed in Curú National Wildlife Refuge in Costa Rica by R. Paul Mansz. This footage plays at full speed and in slow motion. Used with an Open Access license from R. Paul Mansz.

Movie S3. Pileated Woodpeckers that were filmed taking off into cruising flights in the Pearl River swamp in Louisiana by the author. This footage plays at half speed.

Movie S4. Video footage of Northern Mockingbirds in flight that was obtained by the author near the Pearl River swamp in Mississippi.

Movie S5. Video footage of Loggerhead Shrikes in flight that was obtained by the author near the Pearl River swamp in Mississippi.
